# Comparing standard- and low-dose CBCT in diagnosis and treatment decisions for impacted mandibular third molars: a non-inferiority randomised clinical study

**DOI:** 10.1007/s00784-024-06022-5

**Published:** 2024-11-19

**Authors:** Kuo Feng Hung, Andy Wai Kan Yeung, May Chun Mei Wong, Michael M. Bornstein, Yiu Yan Leung

**Affiliations:** 1 Division of Applied Oral Sciences and Community Dental Care, Faculty of Dentistry, The University of Hong Kong, Hong Kong SAR, China; 2 Department of Oral Health & Medicine, University Center for Dental Medicine Basel UZB, University of Basel, Basel, Switzerland; 3 Division of Oral and Maxillofacial Surgery, Faculty of Dentistry, The University of Hong Kong, Hong Kong SAR, China

**Keywords:** Mandibular canal, Mandibular nerve, Radiation protection, Radiation dosage, Clinical decision-making

## Abstract

**Objective:**

This randomised clinical study aimed to assess the influence of low-dose cone-beam computed tomography (CBCT) on the visibility of the mandibular canal (MC) and its proximity to mandibular third molars (M3Ms) as assessed by general dental practitioners (GPs) and oral-maxillofacial surgeons (OMFSs), as well as its impact on their clinical decisions, when compared to standard-dose CBCT.

**Methods:**

154 impacted M3Ms from 90 patients were randomly assigned to three groups for two CBCT exposures using one standard-dose (333 mGy×cm^2^) and one of the three investigated low-dose (78–131 mGy×cm^2^) protocols. Blinded assessments of the MC visibility, M3M-MC proximity, surgical approach, crown/root sectioning, and referral decisions, were made by GPs and OMFSs on the images separately. Pairwise comparisons for MC visibility between paired scans were evaluated using Wilcoxon signed rank test, followed by a non-inferiority test with non-inferiority margin of 0.5 on a four-point scale. Differences in other variables between paired scans were evaluated using Wilcoxon signed-rank or McNemar tests.

**Results:**

The majority (78.5–99.3%) of MCs were clearly identified on standard-dose CBCT by all observers. Pairwise comparisons showed significant differences between paired scans only in MC visibility but not in the M3M-MC proximity or treatment decisions. The mean differences in MC visibility between paired scans ranged 0-0.22 with the upper bounds of the 95% confidence intervals (0.09–0.36) falling within the non-inferiority region.

**Conclusions:**

The investigated low-dose CBCT protocols could provide acceptable image quality for the evaluation of impacted M3Ms in most cases. When compared to standard-dose CBCT, these low-dose CBCT images did not significantly affect the assessments of the M3M-MC proximity, treatment strategies, and patient management decisions made by GPs and OMFSs.

**Clinical relevance:**

The low-dose protocols might be clinically acceptable for M3M management while greatly reducing radiation exposure.

## Introduction

Mandibular third molar (M3M) impaction is a common dental problem globally, affecting approximately 25% of the population [1]. Due to the close proximity between the M3M and the mandibular canal (MC), there is a risk of damaging the inferior alveolar nerve (IAN) during the surgical removal of an impacted M3M [2–4]. Such injuries could cause permanent anaesthesia or dysaesthesia of the ipsilateral lower lip and chin area, resulting in depression and reduced quality of life for affected patients [5, 6].

Periapical radiography, panoramic radiography, and cone-beam computed tomography (CBCT) are imaging techniques frequently used for IAN risk assessment and treatment planning prior to M3M surgery [7, 8]. Due to the higher radiation dose, CBCT imaging should only be applied when a dentist faces an unique clinical question in a specific patient case that cannot be answered through panoramic imaging, as recommended by the European Academy of Dentomaxillofacial Radiology [8]. It has been suggested that CBCT does not significantly change diagnoses, treatment decisions, or patient outcomes in M3M management when compared to panoramic radiography [8]. Even so, many surveys and radiology audits reveal that M3M assessment remains a top reason for CBCT examinations in both hospital and private clinic settings [9–12]. A growing trend of using CBCT for M3M assessment may result from the improved confidence clinicians gain from 3D visualization of the morphology of impacted M3Ms. This enhanced visualization assists clinicians in better understanding the spatial relationship between the MC and M3Ms, preventing them from legal implications arising from postoperative complications [8, 13–15].

Previous non-clinical human skull studies have reported that modifying the acquisition parameters for CBCT imaging can significantly reduce radiation dose while maintaining the visibility of MC [16, 17]. While some low-dose protocols have been suggested, these studies were conducted using human skulls. The absence of soft tissue, presence of collapsed trabecular beams, and decreased intertrabecular spaces in the CBCT images may not adequately represent actual clinical scenarios, and thus these studies have emphasized the need to validate their findings through prospective clinical studies. Additionally, previous studies have primarily focused on the visibility of anatomical landmarks relevant to M3M assessments on low-dose CBCTs, as assessed by radiologists who are not always involved in the diagnosis and treatment decision-making process for M3Ms. Oral-maxillofacial radiology is not universally recognized as a sub-specialty, resulting in varying requirements for CBCT prescription and interpretation across various countries and jurisdictions [18]. Particularly in some Asian and European countries such as China, India, Germany, Austria, Switzerland, and France, and regions like Hong Kong, where oral-maxillofacial radiology is not recognized as a distinct sub-specialty, the regulations for CBCT prescription and interpretation are not firmly established [9, 11, 19]. In these areas, it’s common for general dental practitioners (GPs) and oral-maxillofacial surgeons (OMFSs) to prescribe, perform, and interpret CBCT for dental indications, often without the assistance of registered specialists in oral-maxillofacial radiology. Surveys suggest that dentists, particularly those operating in private clinics, often prescribe CBCT scans without employing a low-dose protocol, indicating a lack of sufficient knowledge about low-dose CBCT [9–11]. This highlights the critical need to investigate the diagnostic efficacy of low-dose CBCT in evaluating MC visibility and its proximity to M3M among both GPs and OMFSs, as well as its impact on their clinical decisions regarding surgical approaches and referral choices when managing impacted M3Ms. Therefore, the objectives of this study were to (1) assess whether using low-dose CBCT images would significantly affect assessments on the visibility of MC and the proximity between MC and M3Ms by GPs and OMFSs, and (2) investigate whether its use would significantly change their clinical decisions, including the surgical approach, the necessity for crown or root sectioning, and referral decisions, as compared to assessments based on standard-dose CBCT images.

## Materials and methods

### Study design

This non-inferiority randomised clinical study was designed following the Reporting of Noninferiority and Equivalence Randomised Trials: Extension of the CONSORT 2010 Statement [20]. The non-inferiority margin for evaluations using a four-point scale was determined at a threshold score of 0.5, as recommended by previous diagnostic imaging studies [21, 22]. Based on the six-level hierarchical model proposed by Fryback and Thornbury for diagnostic imaging studies, this study is at level 4 investigating the image quality of low-dose CBCTs and their impact on clinicians’ diagnostic judgments, the resulting treatment plans, and patient management [23]. In line with previous ethically-approved studies assessing low-dose CT/CBCT [24–27], this study adhered strictly to the guidelines set by the International Commission on Radiological Protection (ICRP) and complied with the radiological protection standards for human research regulations. These regulations highlight the potential benefits at the population level of such projects, aiming to reduce radiation exposure in routine clinical settings [28, 29]. This study has been approved by local institutional review board (UW21-027) and registered on Clinical Trials Registry (HKUCTR-2977).

### Study subjects

#### Inclusion criteria

Patients with an impacted M3M indicated for removal, presenting to Oral and Maxillofacial Surgery, Faculty of Dentistry, The University of Hong Kong from 2021 to 2022, were screened for inclusion. Patients whose panoramic radiographs displayed a high risk of IAN injury, as identified by the presence of root darkening, interruption of the canal wall, diversion of the MC, and an unclear upper MC border where the exact spatial relationship between the M3M roots and the MC could not be determined, were considered eligible. Patients under the age of 18, those with a cystic lesion associated with the M3M, or those with a history of mandibular trauma or radiation therapy were excluded. Written informed consent was obtained from all participating patients.

#### Sample size determination

Based on previous ex-vivo studies [17, 30–32] and our pilot study, the median sensitivity of standard-dose CBCT for identifying the MC at the second/third molar sites was 97.5%. In order to achieve 90% power and a 2.5% one-sided type I error with a non-inferiority margin of 10% [33], fifty-one M3Ms were required for each group. Since three low-dose protocols were investigated in this study, at least 153 M3Ms were required.

#### Randomisation

A randomisation list was prepared by a research assistant (N.S.M.W) to randomly allocate the indicated M3Ms of the patients into three groups. The allocation sequence was concealed in sequentially numbered, opaque and sealed envelopes. After obtaining written informed consent from the patient, the research assistant opened the sealed envelope and informed the radiographer of the corresponding scanning protocols to be used for the patient.

#### Low-dose protocol determination

The three low-dose protocols investigated were determined based on our non-clinical pilot study with twenty M3Ms in dry mandibles from the Human Bone Collection of HKU Faculty of Dentistry [34]. In the pilot study, each M3M had five small field-of-view (FOV) CBCT scans performed with the ProMax 3D Mid (Planmeca Oy, Helsinki, Finland) using one standard-dose and four low-dose protocols. Eventually, one of the four low-dose protocols was excluded due to significantly lower MC visibility compared to paired standard-dose scans. The remaining three low-dose protocols were subsequently investigated in this clinical study (Table 1).

**Table 1:** Acquisition parameters of the standard- and low-dose protocols

Imaging protocol	Group	Default setting	ULD mode	Rotation	FOV (D in cm × H in cm)	Tube voltage (kV)	Tube current (mA)	Exposure time (s)	Voxel size (mm)	Dose in DAP (mGy×cm^2^)	Effective dose in µSv
Low-dose CBCT	1	Low resolution	Off	360*°*	4 × 5	90	5.6	6	0.4	131	28
	2	Normal resolution	On	360*°*	4 × 5	90	5.6	4	0.2	78	18
	3	High resolution	On	360*°*	4 × 5	90	7.1	5	0.15	123	29
Standard-dose CBCT	1, 2, 3	Normal resolution	Off	360*°*	4 × 5	90	8	12	0.2	322	79

*CBCT*, cone-beam computed tomography; *CTDI*, computed tomography dose index; *DAP*, dose-area product; *FOV*, field-of-view; *ULD*, ultra-low dose; *D*, diameter; *H*, height

The effective doses in mSv for the Planmeca ProMax 3D Mid can be found in the following source: https://kaebekirurgiskklinik.dk/wp-content/uploads/2019/10/PlanmecaProMax3DMid-stra%CC%8Aledoser.pdf

#### Image acquisition

The justification of CBCT scans was performed by senior OMFSs, which is a standard practice in this institution. The M3Ms in each group underwent two small FOV CBCT scans performed with the ProMax 3D Mid using one standard-dose (333 mGy×cm^2^) and one of the three investigated low-dose (78–131 mGy×cm^2^) protocols, respectively. The standard-dose protocol used for all M3Ms was the default “Normal-Resolution” setting pre-set by the manufacturer. The low-dose protocols used for M3Ms in Group 1–3 are described in Table 1.

#### Image preparation

**Fig. 1:**
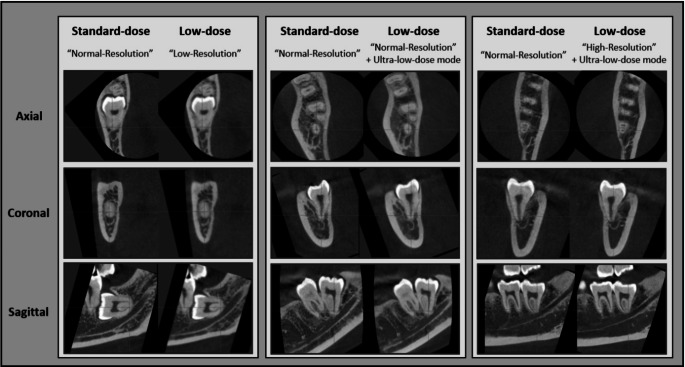
Axial, coronal, and sagittal views of registered paired standard-dose and low-dose CBCT scans. The left, middle, and right pairs display CBCT images acquired using default normal-resolution settings paired with default low-resolution settings, default normal-resolution setting with Ultra-low-dose mode, and default high-resolution setting with Ultra-low-dose mode, respectively

**Fig. 2:**
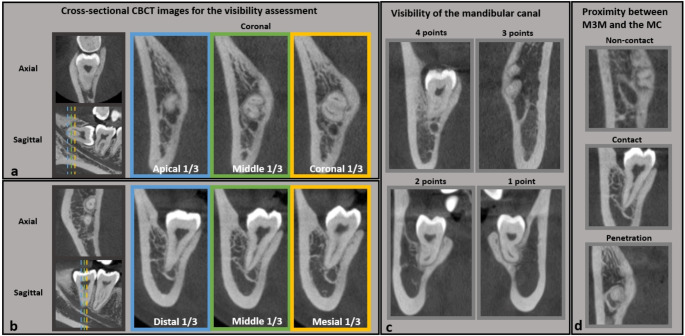
Cross-sectional CBCT images determined for the mandibular third molar (M3M) on each registered scan for image assessment (**a** and **b**). For tilted or horizontally impacted M3Ms, the coronal slices were determined at the apical, middle, and coronal thirds of the M3M root(s) (**a**). For vertically impacted M3Ms, the coronal image slices were determined at the distal, middle, and mesial aspects of the M3M, respectively (**b**). Representative CBCT images displaying varying visibility of the mandibular canal (**c**) and M3M-MC proximity (**d**)

To ensure consistent and reliable image quality assessments for identical slices of paired standard- and low-dose scans, this study adopted a widely-accepted method proposed in previous diagnostic imaging studies [17, 35–41], where specific designated image slices from 3D scans (CT, CBCT, or MRI) were selected for observation. This approach allows image assessments to be conducted on the same pixel-to-pixel level. An operator, who did not participate in the image assessment process, aligned the standard- and low-dose CBCT scans for each patient using ITK-SNAP program (Fig. 1). The operator identified multiple cross-sectional slices, including coronal, sagittal, and axial views from the paired cross-sectional views of each M3M (Fig. 2). For vertically impacted M3Ms, three coronal views were determined at the distal, middle, and mesial aspects of the M3M, respectively. For tilted/horizontally impacted M3Ms, three coronal views were determined at the apical, middle, and coronal thirds of the M3M root(s). The sagittal and axial views that exhibited the shortest distance between the M3M root(s) and the MC and/or passed through the long axis of the M3M were selected. Afterwards, the cross-sectional views were arranged separately for each scan to prepare for the subsequent image assessments. Several variables were assessed on the anonymised cross-sectional CBCT views of the M3M from each scan (i.e., either standard- and low-dose scan) in a randomized sequence to minimize the potential bias in the assessment.

#### Image assessment

Prior to the image assessment, all observers underwent a calibration session using CBCT images that were not included in this study. Four blinded observers, including two GPs and two OMFSs separately assessed the CBCT images. The two GPs are part-time clinical teaching staff while the two OMFSs are senior specialist trainees with over six years of specialty training. All observers have accumulated more than seven years of experience in CBCT diagnosis. The MC visibility, M3M-MC proximity, surgical approach, the necessity for crown or root sectioning, and referral decisions were evaluated for each M3M.

#### The visibility of the MC

For each scan, the MC visibility was assessed on three designated coronal views (Fig. 2), respectively, using a four-point scale adapted from previous studies [16, 17] as follows:

1. Four points for a clearly identifiable MC border (excluding the portion overlapped by the M3M).
2. Three points for a partially identifiable MC border, allowing clear identification of the MC position.
3. Two points for a partially identifiable MC border, not allowing clear identification of the MC position.
4. One point for an unidentifiable MC border and position.

The average MC visibility score for each scan was calculated based on the scores from the three views. Scans with an average score higher 2.5 (the median of the score range) were considered diagnostically acceptable otherwise unacceptable.

#### Proximity between M3M and the MC

The proximity between M3M and the MC was assessed for each M3M based on coronal, sagittal, and axial views. The proximity was categorised based on their spatial relationship (Fig. 2), as proposed in a pre-surgical assessment of M3M removal surgical risk [42].

1. Penetration: M3M apex penetrating the MC border.
2. Contact: M3M apex in contact with the MC border.
3. Non-contact: M3M apex not in contact with the MC border.

#### Treatment decisions

Treatment decisions were made based on coronal, sagittal, and axial views of each M3M. The observers first decided a surgical approach for each M3M, choosing between full removal and coronectomy. The coronectomy technique is defined as the surgical removal of the crown of an M3M while intentionally retaining the root [2]. The definition, indications, and surgical procedures of M3M coronectomy were explained to all observers before assessing the images. For M3Ms decided for full removal, the necessity for crown/root sectioning was assessed. The two GPs were further requested to decide whether they would refer the case to an OMFS.

All observers repeated the assessments on 20% of the cases to evaluate intra-observer reproducibility.

### Statistical analysis

Weighted Kappa (the MC visibility and M3M-MC proximity) and Cohen’s Kappa (surgical approach, crown/root sectioning, and referral decisions) values were calculated to assess intra- and inter-observer agreement on the diagnostic judgments and treatment decisions. The Landis and Koch’s scale was adopted for the interpretation of kappa values, in which values < 0 indicate no agreement, 0-0.20 is slight, 0.21–0.40 is fair, 0.41–0.60 is moderate, 0.61–0.80 is substantial, and 0.81-1 is almost perfect agreement [43]. Pairwise comparisons for the MC visibility between paired standard- and low-dose scans were first evaluated using Wilcoxon signed rank test, followed by a non-inferiority test. In the non-inferiority test, the MC visibility scale at the low-dose scan was considered statistically non-inferior to the scale at the paired standard-dose scan if the upper bounds of the one-sided 95% confidence interval (CI) for the mean differences in visibility scores across all observers were less than the non-inferiority margin of 0.5 scale. Pairwise comparisons for the M3M-MC proximity, surgical approach, crown/root sectioning, and referral decisions between paired scans were evaluated using Wilcoxon signed rank test or McNemar test. The significance level chosen for all of the statistical tests mentioned above was set at 0.05. All analyses were performed using SPSS (Version 28.0, IBM Corp., Armonk, NY, USA).

## Results

Ninety patients (36 males and 54 females) with a mean age of 30.3 years were included in this study. A total of 154 M3Ms from the ninety patients were equally distributed into three groups, resulting in a total of 308 scans. Each M3M had two CBCT exposures using one standard-dose and one of the three investigated low-dose protocols (Table 1), respectively. The intra-observer agreements ranged 0.64–0.83 for the MC visibility, 0.71–0.82 for the M3M-MC proximity, 0.73-1 for surgical approach, 0.79–0.93 for crown/root sectioning, and 0.67–0.81 for referral decisions. As for the inter-observer agreements, it ranged 0.34–0.50 for the MC visibility, 0.53–0.61 for the M3M-MC proximity, 0.52–0.58 for surgical approach, 0.38–0.40 for crown/root sectioning, and 0.59 for referral decisions. Based on the cut-off point at the median of the MC visibility score range, 85.1–99.4% of the standard-dose scans were considered diagnostically acceptable for M3M assessment. The M3M-MC proximity score was higher when assessed by GPs compared to OMFSs, leading the GPs to refer more than half of the cases (64.9–85.7%) to an OMFS. Full removal approach was recommended in most of the cases (66.9–98.1%), with the necessity for crown/root sectioning (68-98.7%) (Table 2).

**Table 2:** The proportions of diagnostic judgment and treatment decisions assessed on standard-dose CBCT images by observers with different qualifications

		GP1	GP2	OMFS1	OMFS2
**Diagnostic judgement**					
MC Visibility	average score < 2	3.9%	3.2%	0.6%	0.6%
	average score ≥ 2 and < 3	17.5%	11.0%	0	7.1%
	average score ≥ 3 and < 4	53.2%	23.4%	13.6%	18.8%
	average score = 4	25.3%	62.3%	85.7%	73.4%
Diagnostic acceptability	Diagnostically acceptable (MC Visibility > 2.5)	85.1%	90.9%	99.4%	95.5%
	Diagnostically unacceptable (MC Visibility ≤ 2.5)	14.9%	9.1%	0.6%	4.5%
M3M-MC proximity	Non-contact	37%	58.4%	56.5%	73.4%
	Contact	39.6%	22.1%	32.5%	24.7%
	Penetration	23.4%	19.5%	11%	1.9%
**Treatment decisions**					
Surgical approach	Full removal	66.9%	77.9%	98.1%	97.4%
	Coronectomy	33.1%	22.1%	1.9%	2.6%
Necessity for crown or root sectioning	Yes	68.0%	75.0%	72.2%	98.7%
	No	32.0%	25.0%	27.8%	1.3%
Referral to an OMFS	Yes	64.9%	85.7%	NA	NA
	No	35.1%	14.3%	NA	NA

*CBCT*, cone-beam computed tomography; *OMFS*, oral-maxillofacial surgeon; *GP*, general dental practitioner; *MC*, mandibular canal; *M3M*, mandibular third molar; *NA*, not applicable

**Fig. 3:**
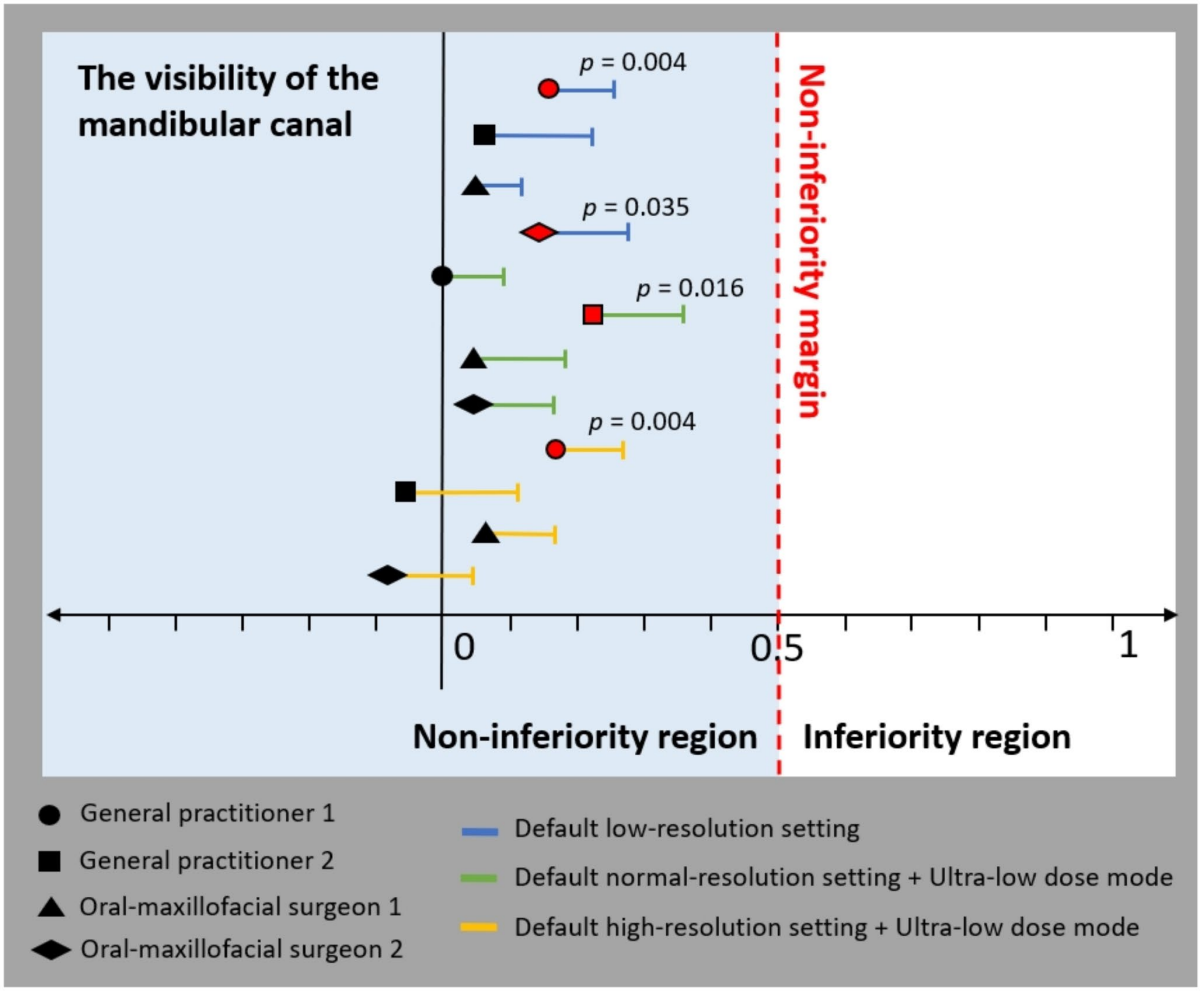
Non-inferiority plot displaying the mean differences in MC visibility scores (black/red shapes) between paired standard- and low-dose scans for each of the three low-dose protocols (blue, green, and yellow) and their upper bounds of the one-sided 95% confidence intervals for all observer (circle, square, triangle, and diamond shapes). Statistically significant *p*-values from the Wilcoxon signed-rank test are provided with shapes in red

Using the same cut-off point, it was observed that 86.3–100%, 78.4–98%, and 80.8–100% of the scans taken using low-dose protocol 1, 2, and 3 respectively, were considered diagnostically acceptable for M3M assessment. Pairwise comparisons exhibited statistically significant differences in MC visibility scores between paired standard- and low-dose scans as evaluated by two GPs and one OMFS when assessing one or two of the three low-dose protocols (Table 3). For all observers, the mean absolute differences in MC visibility between paired scans ranged from 0 to 0.22 score (Table 3). Their upper bounds of the one-sided 95% CIs (0.09–0.36) were all less than the predefined non-inferiority margin of 0.5 score threshold on a four-point scale, falling within the non-inferiority region (Fig. 3). No statistically significant differences were observed in the M3M-MC proximity (*p* values ranging from 0.132 to 0.705), surgical approach (*p* values ranging from 0.453 to 1.000), crown/root sectioning (*p* values ranging from 0.125 to 1.000), and referral decisions (*p* values ranging from 0.250 to 1.000) made by the four observers between paired scans for all three low-dose protocols.

**Table 3:** Comparisons of mandibular canal visibility between paired standard- and low-dose CBCT as assessed by observers with different qualifications

	Visibility of the mandibular canal			
	Mean/Median score (4-point scale)		Mean absolute difference between paired scans (upper bound of one-sided 95% CI)	Wilcoxon Signed Rank test *p* value
	SD scans	LD scans		
*Standard-dose CBCT (normal-resolution) vs. Low-dose CBCT (low-resolution without ULD mode)*				
GP1	3.44 / 4	3.28 / 3	0.16 (0.25)	**0.004**
GP2	3.71 / 4	3.65 / 4	0.06 (0.22)	0.397
OMFS1	3.96 / 4	3.92 / 4	0.04 (0.12)	0.317
OMFS2	3.78 / 4	3.65 / 4	0.14 (0.27)	**0.035**
*Standard-dose CBCT (normal-resolution) vs. Low-dose CBCT (normal-resolution with ULD mode)*				
GP1	3.01 / 3	3.01 / 3	0 (0.09)	1.000
GP2	3.55 / 4	3.33 / 4	0.22 (0.36)	**0.016**
OMFS1	3.8 / 4	3.76 / 4	0.04 (0.18)	0.593
OMFS2	3.67 / 4	3.63 / 4	0.04 (0.16)	0.564
*Standard-dose CBCT (normal-resolution) vs. Low-dose CBCT (high-resolution with ULD mode)*				
GP1	3.28 / 3	3.1 / 3	0.17 (0.27)	**0.004**
GP2	3.48 / 4	3.54 / 4	0.06 (0.11)	0.531
OMFS1	3.9 / 4	3.85 / 4	0.06 (0.17)	0.317
OMFS2	3.71 / 4	3.79 / 4	0.08 (0.04)	0.134

*CBCT*, cone-beam computed tomography; *CI*, confidence interval; *ULD*, ultra-low dose; *OMFS*, oral-maxillofacial surgeon; *GP*, general dental practitioner; *SD*, standard-dose; *LD*, low-dose

Wilcoxon Signed Rank test performed for pairwise comparisons

*p* values ≤ 0.05 in bold

## Discussion

This randomised clinical study assessed the impact of three low-dose CBCT protocols on the MC visibility and its proximity to M3Ms. This study also investigated the influence of these low-dose CBCT images on the clinicians’ decisions, including the surgical approach, the necessity for crown or root sectioning, and referral decisions. The results showed significant differences between paired standard- and low-dose scans only in the MC visibility but not in the M3M-MC proximity, surgical approach, crown/root sectioning, and referral decisions. Although some differences in the MC visibility between paired scans were found to be statistically significant in the pairwise comparisons for some observers, the magnitude of these differences was minimal with mean absolute differences ranging from 0 to 0.22 score on a four-point scale. In addition to pairwise comparisons, this study also performed the non-inferiority analysis that has been widely used to compare the image quality of standard- and low-dose diagnostic images, determining whether the image quality of low-dose images is not significantly worse than that of standard-dose images by a predefined, clinically acceptable margin. In this study, the non-inferiority analysis revealed that the upper bounds of the 95% CIs for these mean differences across all observers were significantly below the predefined non-inferiority margin, suggesting that the MC visibility score on the low-dose scan could be considered non-inferior to the score on the paired standard-dose CBCT images.

Previous non-clinical studies using human skulls have demonstrated that the use of pre-programmed low-dose protocols, such as “Ultra-low-dose” or “partial rotation” imaging modes, or manually adjusting acquisition parameters can effectively reduce the dose of CBCT examinations without significantly compromising image quality for assessing the MC. Using these strategies can decrease the number of X-ray photons reaching the sensor, which in turn reduces the radiation dose. However, this may also compromise the image clarity and overall quality by amplifying image noise. Zaki et al. and Tadinada et al. found no significant differences in the MC visibility between full- and half-arc CBCT, resulting in 97.5% sensitivity for full-arc and 95.5% for half-arc rotation protocols [17, 30]. Liljeholm et al. observed a considerable reduction in MC visibility when a full rotation (360°) protocol was applied with a tube voltage of 90 kVp, tube current of 5.6 mA, FOV of 8 × 8 cm, and voxel size of 0.4 mm [16]. Neves et al. [44] and Waltrick et al. [31] recommended different low-dose protocols with reduced tube current and voxel size. A recent clinical study by Cederhag et al. involved 48 patients with impacted M3Ms. Each patient underwent two CBCT scans, one using a default protocol and another using a low-dose protocol with reduced tube current. Their study found no significant differences between the two protocols in terms of the MC visibility and the M3M-MC proximity except the periodontal ligament [25]. However, these findings were based on the assessments by radiologists, who are not always involved in the diagnosis and treatment decision-making process for M3Ms. Importantly, none of these studies included GPs and OMFSs in the image assessment. In certain Asian and European countries, including China, India, Germany, Austria, Switzerland, France, and regions like Hong Kong, it is a common practice for GPs and OMFSs to prescribe, perform, and interpret CBCT for dental indications without the assistance of registered oral-maxillofacial radiologists [9, 11, 19]. From a clinical perspective, it is crucial to assess whether the image quality of low-dose CBCTs would significantly affect clinicians’ decision-making processes. In addition to previous studies, this study investigated the differences not only in the MC visibility and the M3M-MC proximity, but also in the surgical approach, crown/root sectioning, and referral decisions made by GPs and OMFSs between standard- and low-dose CBCT images. Our results revealed that GPs tended to be more conservative in assessing the M3M-MC proximity compared to OMFSs. However, no significant differences were observed in M3M-MC proximity, treatment strategies, and referral decisions between paired scans for all three investigated low-dose protocols. These findings suggest that using the investigated low-dose CBCT protocols for assessing impacted M3Ms may not significantly impact the case difficulty assessment or subsequent treatment decisions by clinicians with different qualifications, compared to standard-dose scans.

The three low-dose protocols exhibit different exposure settings, specifically in terms of voxel sizes and mAs. The voxel size of the standard-dose images is 0.2 mm, while for the low-dose protocols 1, 2, and 3, the voxel sizes are 0.4 mm, 0.2 mm, and 0.15 mm, respectively. Theoretically, a larger voxel may compromise the image quality and thus reduce the accuracy in evaluating intricate anatomical structures. Previous studies suggest a voxel size of less than 0.4 mm for improved visibility of the structural characteristics of the trabecular bone and the MC [16, 17, 31, 35, 44]. More specifically, a voxel size of 0.2 mm has been recommended for better MC assessment [31]. In this study, lower MC visibility was noted in pair-wise comparisons by more observers in low-dose protocol 1 compared to low-dose protocols 2 and 3. This suggests that the larger voxel size in low-dose protocol 1 could potentially account for lower MC visibility, implying that low-dose protocols 2 and 3 may be more reliable options for superior MC visualization. Furthermore, previous studies have demonstrated that the mAs setting has a linear effect on the accuracy of anatomical landmark assessment in the posterior mandible, with higher mA providing better MC visualization [44]. A product of tube current and exposure time of approximately 100 has been recommended for M3M assessment, which is similar to the mAs of the standard-dose protocol used in this study. However, the mAs of the low-dose protocols 2 and 3 was nearly one-third that of the standard-dose protocol, which could lead to degraded image quality and could potentially explain the observed lower MC visibility in images taken using low-dose protocols 2 and 3 as compared to those taken with the standard-dose protocol. Nevertheless, due to the comparable mAs values of low-dose protocols 2 and 3, the impact of mAs difference on the MC visibility might not be significant. Low-dose protocol 2, which has the lowest radiation dose among the other low-dose protocols and the smallest median of the mean absolute MC visibility score difference between paired scans across observers, might be the most optimal choice for M3M assessment.

In daily dental practice, impacted M3Ms are often assessed with the help of panoramic and CBCT images. Although it has been suggested that CBCT does not significantly alter diagnoses, treatment decisions, or patient outcomes in M3M management compared to panoramic radiography [8], M3M assessment remains a top reason for CBCT examinations in both hospital and private clinic settings with a growing trend [9–11]. This could be attributed to the enhanced confidence clinicians gain from 3D visualization of the morphology of impacted M3Ms in determining the spatial relationship between the MC and M3Ms, which can prevent them from legal implications arising from postoperative permanent anaesthesia or dysaesthesia of the lower lip and chin area [8, 13–15]. The effective dose of the CBCT scans using the investigated low-dose protocols ranged from18-29 µSv, which is less than half the dose of the standard-dose scans (79 µSv) and falls within the dose range of panoramic radiography (3.85-38 µSv) [45, 46]. While this study found statistically significant differences in the MC visibility between standard- and low-dose scans, no significant differences were observed in risk assessment, treatment plans, or referral decisions. These findings suggest that although the image quality of the low-dose scans might be slightly inferior in terms of the MC visibility, it could still be sufficient for managing impacted M3Ms. In line with As Long As Diagnostically Acceptable (ALADA) principle, the use of the investigated low-dose protocols might be considered acceptable as they could provide sufficient information for clinical decision-making while minimizing radiation exposure.

Although this study has obtained approval from the local institutional review board and informed consent from the participants, the ethical considerations associated with this study cannot be overlooked. The international radiation protection authorities, such as ICRP, have established ethical regulations on human research involving radiation, highlighting that research projects requiring additional radiation can be justified if they have potential benefits at a population level over the possible individual harm from the extra radiation [28, 29]. In accordance with such regulations, several previous studies involving extra radiation have evaluated low-dose protocols of CT/CBCT for various clinical indications [24–27]. The clinical investigation of these low-dose protocols could potentially lead to a future reduction in radiation dose at the population level. This study was designed to enhance the understanding of the image quality of low-dose CBCT for M3M assessment and its impact on clinical decisions. The findings from this study have potential societal benefits, including a reduction in the radiation dose of CBCT performed for M3M assessments in routine clinical settings. This could be beneficial for a large number of young patients who are more susceptible to ionizing radiation and have a longer post-exposure lifetime. Therefore, at a population level, the potential benefits of this study could be considered to outweigh the possible individual harm.

The use of 3D imaging helps clinicians and patients to make clinical decisions and informed consent in the assessment of the surgical risk of inferior alveolar nerve injury when an impacted M3M has to be removed [47, 48]. The decision lies either to perform total removal or coronectomy of the M3M, depending on the true proximity of the M3M root(s) to the nerve. Inferior alveolar nerve injury could be inevitable even in the best hands if the nerve is in touch with the M3M root during the tooth elevation, leading to neurosensory deficit of the lower lip on the affected side. Although majority of the injuries could resolve completely, a portion may be permanent despite various treatment [49]. Coronectomy, which removes only the M3M’s crown and leaves the root behind, has been proven to prevent inferior alveolar nerve injury in the high-risk cases [50–53]. Despite a small percentage of root migration leading to exposure and re-operation, it is safe in long term [54–56], and thus is a valuable alternative to totally removal of M3M in particular in medium to high-risk cases. With the investigated low-dose protocols, the concerns regarding high radiation doses in routine CBCT could be reduced. The findings of this study might lay down evidence advocating for the use of low-dose CBCT for impacted M3Ms, therefore improving patient radiation safety and protection.

This study has some limitations. First, this study only compared the default normal-resolution setting to default settings with or without an ultra-low dose mode. No manual tube current adjustment was performed. It has been suggested that many default settings for CBCT scans could be further optimised by reducing tube current. The necessity of decreasing tube current for the investigated low-dose protocols should be further evaluated. Furthermore, it’s important to note that in this study, the prescription of CBCT imaging for impacted M3Ms was made by senior OMFSs. This practice may differ from that of radiologists and could potentially influence the study’s results. The potential discrepancies in CBCT examination prescriptions and interpretations among clinicians with different qualifications is an area that warrants further investigation to standardize practices, which is particularly crucial in regions where it is a common practice for GPs and OMFSs to prescribe, perform, and interpret CBCT for dental indications without the assistance of oral-maxillofacial radiologists. Moreover, the image assessment approach used in this study did not allow observers to assess the MC visibility on all slices, hence not fully replicating actual clinical practice. In diagnostic imaging studies using 3D images, the assessment methods commonly used are either scrolling through 3D images or evaluating selected image slices. While the former method is closer to clinical practice, it could inevitably introduce bias when choosing slices for MC visibility assessment. The MC visibility changes along its course from the posterior to the anterior region, and its borders may not consistently present as fully corticated on neighbouring image slices. Consistent with previous studies on image quality assessment, this study first aligned the standard- and low-dose CBCT volumes, and subsequently selected multiple image slices that displayed the closest proximity between the MC and the M3M apex from distal to mesial along the MC course. Despite its inability to evaluate the MC visibility on all slices, this method could still provide observers with a clear understanding of the MC course direction and the spatial relationship between the M3M apex and the MC. Future studies using various image quality assessment methods would be necessary to contribute to a more comprehensive understanding of the differences in image quality between standard- and low-dose CBCT for M3M assessment.

## Conclusion

The investigated low-dose CBCT protocols could provide acceptable image quality for the evaluation of impacted M3Ms in most cases. When compared to standard-dose CBCT, these low-dose CBCT images did not significantly affect the assessments of the M3M-MC proximity, treatment strategies, and patient management decisions made by GPs and OMFSs.

## Data Availability

No datasets were generated or analysed during the current study.
